# [(–)-(1*S*,2*S*)-*N*,*N*′-Bis(2-oxidobenzyl­idene)-1,2-diphenyl­ethane-1,2-diamine]­bis­(pyridine)cobalt(III) perchlorate methanol hemisolvate hemihydrate

**DOI:** 10.1107/S1600536808031887

**Published:** 2008-10-09

**Authors:** Yu-Ting Chen

**Affiliations:** a Department of Chemistry, Dezhou University, Dezhou 253023, People’s Republic of China

## Abstract

In the title compound, [Co(C_28_H_22_N_2_O_2_)(C_5_H_5_N)_2_]ClO_4_·0.5CH_4_O·0.5H_2_O, each Co^III^ ion is coordinated by the tetra­dentate *N*,*N*′-bis­(2-oxidobenzyl­idene)-1,2-diphenyl­ethane-1,2-diamine ligand [Co—N = 1.900 (3) and 1.903 (3) Å; Co—O = 1.885 (3) and 1.891 (3) Å] and two pyridine ligands [Co—N = 1.967 (4) and 1.977 (3) Å] in a distorted octa­hedral geometry. The packing of the cations and anions forms voids of 258 Å^3^, which are filled by methanol and solvent water mol­ecules with half occupancies. O—H⋯O hydrogen bonds between solvent molecules, perchlorate anions and water molecules, and between water molecules and O atoms of the ligand, help to consolidate the crystal packing.

## Related literature

For related crystal structures, see: Korendovych & Rybak-Akimova (2003 ▶); Shi *et al.* (1995 ▶). For general background, see: Amirnasr *et al.* (2001 ▶); Botteher *et al.*, 1997 ▶; Cmi *et al.* (1998 ▶); Henson *et al.* (1999 ▶); Polson *et al.* (1997 ▶); Yamada (1999 ▶); Zhang *et al.* (1990 ▶).
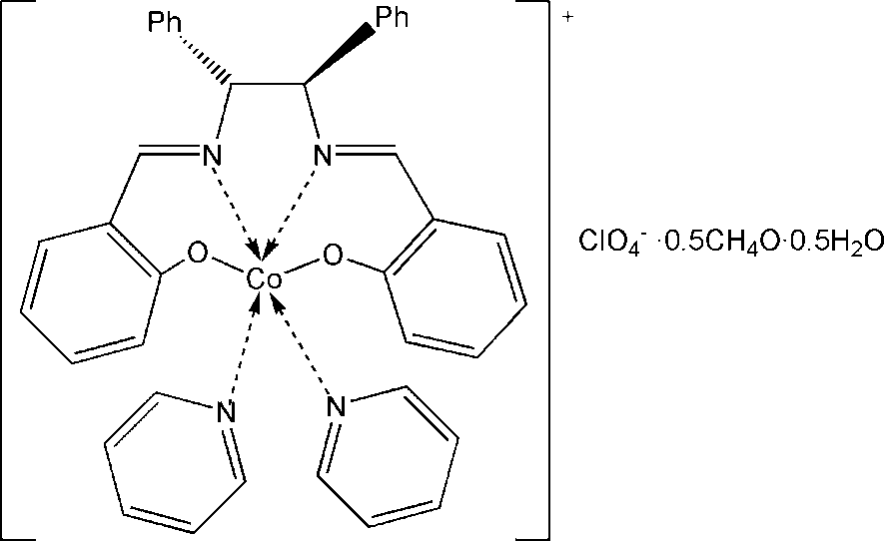


## Experimental

### 

#### Crystal data

[Co(C_28_H_22_N_2_O_2_)(C_5_H_5_N)_2_]ClO_4_·0.5CH_4_O·0.5H_2_O
*M* *_r_* = 760.09
Orthorhombic, 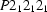

*a* = 10.8900 (3) Å
*b* = 18.6219 (5) Å
*c* = 18.6557 (6) Å
*V* = 3783.24 (19) Å^3^
*Z* = 4
Mo *K*α radiation
μ = 0.58 mm^−1^
*T* = 273 (2) K
0.18 × 0.16 × 0.14 mm

#### Data collection

Bruker APEXII CCD area-detector diffractometer
Absorption correction: multi-scan (*SADABS*; Sheldrick, 2003 ▶) *T*_min_ = 0.903, *T*_max_ = 0.924
42911 measured reflections
7407 independent reflections
5476 reflections with *I* > 2σ(*I*)
*R*_int_ = 0.064

#### Refinement

*R*[*F* ^2^ > 2σ(*F* ^2^)] = 0.050
*wR*(*F* ^2^) = 0.145
*S* = 1.02
7407 reflections
463 parameters
13 restraints
H-atom parameters constrained
Δρ_max_ = 0.48 e Å^−3^
Δρ_min_ = −0.37 e Å^−3^
Absolute structure: Flack (1983 ▶), with 3248 Friedel pairs
Flack parameter: 0.03 (2)

### 

Data collection: *APEX2* (Bruker, 2004 ▶); cell refinement: *SAINT-Plus* (Bruker, 2001 ▶); data reduction: *SAINT-Plus*; program(s) used to solve structure: *SHELXS97* (Sheldrick, 2008 ▶); program(s) used to refine structure: *SHELXL97* (Sheldrick, 2008 ▶); molecular graphics: *XP* (Sheldrick, 1998 ▶); software used to prepare material for publication: *XP*.

## Supplementary Material

Crystal structure: contains datablocks global, I. DOI: 10.1107/S1600536808031887/cv2457sup1.cif

Structure factors: contains datablocks I. DOI: 10.1107/S1600536808031887/cv2457Isup2.hkl

Additional supplementary materials:  crystallographic information; 3D view; checkCIF report

## Figures and Tables

**Table 1:** Hydrogen-bond geometry (Å, °)

*D*—H⋯*A*	*D*—H	H⋯*A*	*D*⋯*A*	*D*—H⋯*A*
O8—H8*D*⋯O6^i^	0.85	1.98	2.831 (14)	178
O8—H8*C*⋯O7	0.85	1.96	2.807 (19)	177
O7—H7⋯O2	0.82	2.08	2.897 (11)	171

Symmetry code: (i) 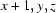.

**Fig. 1.**
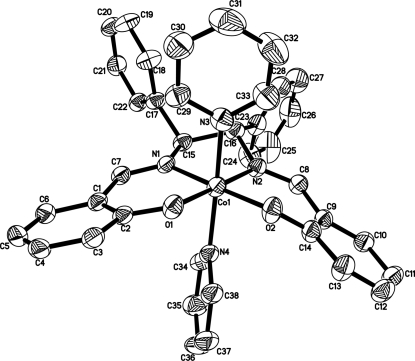
A view of the cation of the title compound with the atom-labelling scheme. Displacement ellipsoids are drawn at the 30% probability level. H atoms omited for clarity.

## supplementary crystallographic information

### Comment

The cobalt complexes with tetradentate Schiff base ligands have been extensively studied due to their important utilities in mimic cobalamin (B_12_) coenzymes (Amirnasr *et al.*, 2001; Cmi *et al.*, 1998; Polson *et al.*, 1997), and as dioxygen carriers and oxygen activators (Yamada, 1999; Henson *et al.*, 1999) . In addition, Co^III^ Schiff base complexes have also been used as antimicrobial agents when their two axial positions are occupied by two amine ligands (Botteher *et al.*, 1997). Herein, we report the new Co^III^ complex based on the chiral tetradentate Schiff base ligand (-)-(1*S*,2S)-*N*,*N*'-Bis(salicylidene)-1,2- diphenyl-1,2-ethanediamine (L), whose structure has been reported recently (Korendovych & Rybak-Akimova, 2003).

In the cation (Fig. 1), the coordination sphere of Co^III^ ion is a distorted octahedron, in which four equational positions come from two N atoms and two O atoms of the tetradentate Schiff base ligand and the apical positions  are occupied by N atoms of two pyridine molecules. The bond lengths of Co—O(L) and Co—N(L) are 1.885 (3), 1.891 (3)A% and 1.900 (3), 1.903 (3)A%, respectively, which are in agreement with the corresponding bond lengths in the similar Co^III^ Schiff base complex *trans*-[Co(salen)(py)_2_][BPh_4_] (Shi *et al.*, 1995)). The distances of Co—N_py_ 1.967 (4) and 1.977 (3)A% are also consistent with those distances in the same complex, but slightly longer than the distances of Co—N_Schiff base_.

### Experimental

The free Schiff base ligand L was synthesized according to the literature (Zhang *et al.*, 1990). The synthsis of the title complex was carried out by reacting CoClO_4_.6H_2_O, pyridine and L (molar ratio 1:2:1 in methanol. After the stirring process was continued for about 30 min at room temperature, the mixture was filtered and the filtrate was allowed to partial evaporate in air for several days to produce crystals suitable for X-ray diffraction. Anal. Calcd for C_38.5_H_35_ClCoN_4_O_7_: C, 60.84; H, 4.64; N, 7.37. Found: C, 60.64; H, 4.65; N, 7.39.

### Refinement

The occupancies of methanol (O7, C39) and crystalline water (O8) molecules were set to 0.5 and not refined. The common U_iso_ was refined for O7 and C39 atoms (methanol). Atom O8 was also refined isotropically. All H atoms were placed in idealized positions (C—H 0.93-0.98 Å; O-H 0.82-0.85 Å), and refined as riding with *U*_iso_(H) = 1.2-1.5*U*_eq_ of the parent atom.

### Crystal data

**Table:**

[Co(C_28_H_22_N_2_O_2_)(C_5_H_5_N)_2_]ClO_4_·0.5CH_4_O·0.5H_2_O	*D*_x_ = 1.334 Mg m^−^^3^*D*_m_ = 1.334 Mg m^−^^3^*D*_m_ measured by not measured
*M**_r_* = 760.09	Mo *K*α radiation, λ = 0.71073 Å
Orthorhombic, *P*2_1_2_1_2_1_	Cell parameters from 8558 reflections
*a* = 10.8900 (3) Å	θ = 2.4–20.8°
*b* = 18.6219 (5) Å	µ = 0.58 mm^−^^1^
*c* = 18.6557 (6) Å	*T* = 273 K
*V* = 3783.24 (19) Å^3^	Block, red-brown
*Z* = 4	0.18 × 0.16 × 0.14 mm
*F*(000) = 1576	

### Data collection

**Table:**

Bruker APEXII CCD area-detector diffractometer	7407 independent reflections
Radiation source: fine-focus sealed tube	5476 reflections with *I* > 2σ(*I*)
graphite	*R*_int_ = 0.064
φ and ω scans	θ_max_ = 26.0°, θ_min_ = 2.2°
Absorption correction: multi-scan (SADABS; Sheldrick, 2003)	*h* = −13→13
*T*_min_ = 0.903, *T*_max_ = 0.924	*k* = −22→22
42911 measured reflections	*l* = −23→23

### Refinement

**Table:**

Refinement on *F*^2^	Hydrogen site location: inferred from neighbouring sites
Least-squares matrix: full	H-atom parameters constrained
*R*[*F*^2^ > 2σ(*F*^2^)] = 0.050	*w* = 1/[σ^2^(*F*_o_^2^) + (0.0885*P*)^2^] where *P* = (*F*_o_^2^ + 2*F*_c_^2^)/3
*wR*(*F*^2^) = 0.145	(Δ/σ)_max_ = 0.001
*S* = 1.02	Δρ_max_ = 0.48 e Å^−^^3^
7407 reflections	Δρ_min_ = −0.37 e Å^−^^3^
463 parameters	Extinction correction: SHELXL97 (Sheldrick, 2008), Fc^*^=kFc[1+0.001xFc^2^λ^3^/sin(2θ)]^-1/4^
13 restraints	Extinction coefficient: 0.0014 (5)
Primary atom site location: structure-invariant direct methods	Absolute structure: Flack (1983), 3248 Friedel pairs
Secondary atom site location: difference Fourier map	Flack parameter: 0.03 (2)

### Special details

**Table:**

Geometry. All e.s.d.'s (except the e.s.d. in the dihedral angle between two l.s. planes) are estimated using the full covariance matrix. The cell e.s.d.'s are taken into account individually in the estimation of e.s.d.'s in distances, angles and torsion angles; correlations between e.s.d.'s in cell parameters are only used when they are defined by crystal symmetry. An approximate (isotropic) treatment of cell e.s.d.'s is used for estimating e.s.d.'s involving l.s. planes.
Refinement. Refinement of *F*^2^ against ALL reflections. The weighted *R*-factor *wR* and goodness of fit *S* are based on *F*^2^, conventional *R*-factors *R* are based on *F*, with *F* set to zero for negative *F*^2^. The threshold expression of *F*^2^ > σ(*F*^2^) is used only for calculating *R*-factors(gt) *etc*. and is not relevant to the choice of reflections for refinement. *R*-factors based on *F*^2^ are statistically about twice as large as those based on *F*, and *R*- factors based on ALL data will be even larger.

### Fractional atomic coordinates and isotropic or equivalent isotropic displacement parameters (Å^2^)

**Table:**

	*x*	*y*	*z*	*U*_iso_*/*U*_eq_	Occ. (<1)
Co1	0.50476 (4)	0.57650 (2)	0.75640 (2)	0.04751 (16)	
Cl1	0.10093 (14)	0.50610 (10)	0.55336 (8)	0.1022 (5)	
O1	0.6076 (3)	0.65694 (14)	0.76901 (15)	0.0568 (7)	
O2	0.5983 (3)	0.55521 (14)	0.67367 (15)	0.0619 (7)	
O3	0.1244 (5)	0.5541 (3)	0.4915 (2)	0.1324 (18)	
O4	0.2142 (4)	0.4859 (3)	0.5844 (3)	0.1202 (15)	
O5	0.0367 (5)	0.4459 (4)	0.5318 (4)	0.175 (3)	
O6	0.0303 (5)	0.5444 (3)	0.6048 (3)	0.1398 (18)	
O7	0.6798 (12)	0.6643 (6)	0.5737 (6)	0.153 (4)*	0.50
H7	0.6495	0.6359	0.6022	0.230*	0.50
O8	0.9347 (13)	0.6770 (6)	0.5559 (7)	0.163 (4)*	0.50
H8C	0.8572	0.6746	0.5606	0.195*	0.50
H8D	0.9657	0.6377	0.5703	0.195*	0.50
N1	0.4061 (3)	0.59406 (15)	0.83873 (17)	0.0460 (7)	
N2	0.3989 (3)	0.49659 (15)	0.74280 (16)	0.0462 (7)	
N3	0.6234 (3)	0.52046 (17)	0.81394 (17)	0.0506 (8)	
N4	0.4010 (4)	0.63945 (18)	0.6973 (2)	0.0601 (9)	
C1	0.5455 (4)	0.68095 (18)	0.8912 (2)	0.0498 (9)	
C2	0.6202 (4)	0.69134 (19)	0.8297 (2)	0.0497 (9)	
C3	0.7149 (4)	0.7448 (2)	0.8337 (3)	0.0571 (10)	
H3	0.7637	0.7540	0.7938	0.069*	
C4	0.7341 (4)	0.7824 (2)	0.8957 (3)	0.0607 (11)	
H4	0.7955	0.8171	0.8973	0.073*	
C5	0.6637 (4)	0.7697 (2)	0.9561 (3)	0.0632 (11)	
H5	0.6799	0.7947	0.9982	0.076*	
C6	0.5700 (4)	0.7204 (2)	0.9542 (2)	0.0578 (10)	
H6	0.5222	0.7129	0.9948	0.069*	
C7	0.4382 (4)	0.63562 (19)	0.8909 (2)	0.0477 (9)	
H7A	0.3883	0.6364	0.9313	0.057*	
C8	0.5417 (3)	0.4304 (2)	0.6702 (2)	0.0482 (8)	
C9	0.5718 (4)	0.3609 (2)	0.6450 (2)	0.0578 (10)	
H9	0.5178	0.3231	0.6536	0.069*	
C10	0.6775 (4)	0.3479 (2)	0.6087 (3)	0.0666 (12)	
H10	0.6975	0.3016	0.5941	0.080*	
C11	0.7546 (4)	0.4046 (3)	0.5938 (3)	0.0729 (13)	
H11	0.8273	0.3959	0.5692	0.087*	
C12	0.7280 (4)	0.4727 (3)	0.6140 (3)	0.0688 (12)	
H12	0.7809	0.5098	0.6013	0.083*	
C13	0.6209 (4)	0.4882 (2)	0.6542 (2)	0.0539 (10)	
C14	0.4274 (3)	0.4402 (2)	0.7069 (2)	0.0493 (9)	
H14	0.3701	0.4033	0.7045	0.059*	
C15	0.2981 (3)	0.5456 (2)	0.8473 (2)	0.0473 (9)	
H15	0.3202	0.5088	0.8826	0.057*	
C16	0.2763 (3)	0.5075 (2)	0.7757 (2)	0.0486 (9)	
H16	0.2305	0.5406	0.7448	0.058*	
C17	0.1814 (3)	0.5823 (2)	0.8738 (2)	0.0510 (9)	
C18	0.1280 (4)	0.6393 (3)	0.8389 (3)	0.0713 (12)	
H18	0.1664	0.6588	0.7989	0.086*	
C19	0.0192 (4)	0.6679 (3)	0.8621 (3)	0.0758 (13)	
H19	−0.0159	0.7057	0.8367	0.091*	
C20	−0.0378 (4)	0.6424 (2)	0.9208 (3)	0.0686 (12)	
H20	−0.1112	0.6629	0.9361	0.082*	
C21	0.0128 (4)	0.5855 (2)	0.9584 (2)	0.0649 (11)	
H21	−0.0263	0.5672	0.9988	0.078*	
C22	0.1243 (4)	0.5560 (2)	0.9345 (2)	0.0536 (9)	
H22	0.1601	0.5183	0.9598	0.064*	
C23	0.1990 (4)	0.4396 (2)	0.7842 (2)	0.0571 (10)	
C24	0.2292 (6)	0.3877 (3)	0.8359 (3)	0.0832 (15)	
H24	0.2987	0.3929	0.8644	0.100*	
C25	0.1518 (7)	0.3278 (3)	0.8433 (4)	0.105 (2)	
H25	0.1708	0.2934	0.8776	0.126*	
C26	0.0507 (6)	0.3185 (4)	0.8023 (4)	0.1008 (18)	
H26	0.0012	0.2783	0.8083	0.121*	
C27	0.0227 (5)	0.3690 (4)	0.7522 (4)	0.1023 (19)	
H27	−0.0467	0.3632	0.7237	0.123*	
C28	0.0970 (4)	0.4298 (3)	0.7429 (3)	0.0739 (12)	
H28	0.0765	0.4637	0.7084	0.089*	
C29	0.5918 (4)	0.4749 (2)	0.8660 (3)	0.0630 (11)	
H29	0.5086	0.4681	0.8749	0.076*	
C30	0.6749 (5)	0.4372 (3)	0.9074 (3)	0.0778 (13)	
H30	0.6486	0.4067	0.9437	0.093*	
C31	0.7970 (5)	0.4464 (4)	0.8932 (4)	0.0970 (19)	
H31	0.8562	0.4212	0.9188	0.116*	
C32	0.8306 (4)	0.4940 (3)	0.8399 (4)	0.0849 (16)	
H32	0.9133	0.5017	0.8301	0.102*	
C33	0.7431 (4)	0.5298 (2)	0.8013 (3)	0.0631 (11)	
H33	0.7675	0.5615	0.7654	0.076*	
C34	0.3626 (6)	0.6221 (3)	0.6330 (3)	0.0879 (17)	
H34	0.3810	0.5764	0.6160	0.105*	
C35	0.2964 (7)	0.6674 (3)	0.5888 (4)	0.114 (3)	
H35	0.2703	0.6521	0.5438	0.137*	
C36	0.2701 (7)	0.7351 (4)	0.6125 (4)	0.117 (2)	
H36	0.2287	0.7672	0.5829	0.140*	
C37	0.3057 (6)	0.7558 (3)	0.6813 (4)	0.0915 (17)	
H37	0.2856	0.8008	0.6996	0.110*	
C38	0.3721 (5)	0.7068 (2)	0.7213 (3)	0.0691 (12)	
H38	0.3985	0.7203	0.7668	0.083*	
C39	0.5940 (19)	0.7285 (10)	0.5644 (10)	0.153 (4)*	0.50
H39A	0.5563	0.7398	0.6095	0.230*	0.50
H39B	0.5316	0.7170	0.5299	0.230*	0.50
H39C	0.6403	0.7692	0.5479	0.230*	0.50

### Atomic displacement parameters (Å^2^)

**Table:**

	*U*^11^	*U*^22^	*U*^33^	*U*^12^	*U*^13^	*U*^23^
Co1	0.0420 (3)	0.0414 (3)	0.0591 (3)	−0.0055 (2)	0.0071 (3)	−0.0055 (2)
Cl1	0.0737 (9)	0.1434 (14)	0.0895 (9)	−0.0270 (9)	0.0163 (7)	−0.0380 (9)
O1	0.0533 (16)	0.0459 (14)	0.0712 (18)	−0.0099 (12)	0.0084 (14)	−0.0100 (12)
O2	0.0624 (17)	0.0527 (16)	0.0705 (17)	−0.0156 (14)	0.0165 (15)	−0.0143 (13)
O3	0.125 (4)	0.198 (5)	0.073 (2)	−0.016 (4)	0.013 (2)	−0.008 (3)
O4	0.073 (3)	0.155 (4)	0.132 (3)	−0.013 (3)	0.003 (3)	−0.009 (3)
O5	0.109 (4)	0.213 (6)	0.204 (6)	−0.068 (4)	0.018 (4)	−0.090 (5)
O6	0.141 (4)	0.157 (4)	0.121 (3)	0.013 (4)	0.047 (3)	−0.035 (3)
N1	0.0350 (15)	0.0403 (16)	0.0626 (18)	0.0018 (13)	0.0020 (14)	−0.0051 (14)
N2	0.0385 (15)	0.0437 (16)	0.0564 (17)	−0.0024 (13)	0.0045 (15)	−0.0035 (14)
N3	0.0392 (17)	0.0451 (17)	0.068 (2)	−0.0015 (14)	−0.0004 (15)	−0.0106 (15)
N4	0.060 (2)	0.0524 (19)	0.068 (2)	−0.0124 (18)	−0.0020 (19)	0.0029 (16)
C1	0.044 (2)	0.0363 (19)	0.070 (2)	0.0064 (16)	−0.0002 (18)	−0.0026 (17)
C2	0.041 (2)	0.0355 (19)	0.073 (3)	0.0044 (16)	−0.0035 (19)	−0.0068 (18)
C3	0.044 (2)	0.047 (2)	0.081 (3)	0.0023 (18)	−0.003 (2)	−0.003 (2)
C4	0.047 (2)	0.040 (2)	0.096 (3)	0.0008 (18)	−0.018 (2)	−0.010 (2)
C5	0.054 (3)	0.053 (2)	0.083 (3)	0.002 (2)	−0.016 (2)	−0.014 (2)
C6	0.057 (3)	0.046 (2)	0.070 (3)	0.0029 (19)	−0.009 (2)	−0.0085 (19)
C7	0.046 (2)	0.0396 (18)	0.058 (2)	0.0036 (17)	0.0047 (18)	−0.0028 (17)
C8	0.0429 (19)	0.0465 (19)	0.0552 (19)	−0.0043 (17)	0.0000 (15)	−0.0079 (18)
C9	0.050 (2)	0.052 (2)	0.071 (3)	−0.0026 (19)	0.002 (2)	−0.0113 (19)
C10	0.052 (2)	0.062 (3)	0.086 (3)	0.003 (2)	0.004 (2)	−0.017 (2)
C11	0.046 (3)	0.082 (3)	0.091 (3)	−0.001 (2)	0.014 (2)	−0.027 (3)
C12	0.050 (2)	0.074 (3)	0.082 (3)	−0.015 (2)	0.018 (2)	−0.015 (2)
C13	0.049 (2)	0.055 (2)	0.057 (2)	−0.0091 (19)	0.0057 (18)	−0.0100 (18)
C14	0.045 (2)	0.043 (2)	0.060 (2)	−0.0073 (17)	0.0032 (18)	−0.0049 (17)
C15	0.039 (2)	0.045 (2)	0.058 (2)	0.0004 (16)	0.0063 (17)	0.0015 (17)
C16	0.0388 (18)	0.045 (2)	0.062 (2)	−0.0017 (17)	0.0045 (16)	−0.0038 (17)
C17	0.0359 (18)	0.052 (2)	0.065 (2)	0.0015 (17)	0.0010 (17)	−0.0017 (19)
C18	0.059 (3)	0.070 (3)	0.085 (3)	0.015 (2)	0.010 (2)	0.009 (2)
C19	0.054 (3)	0.075 (3)	0.098 (3)	0.027 (2)	0.008 (3)	0.006 (2)
C20	0.049 (2)	0.068 (3)	0.089 (3)	0.011 (2)	0.004 (2)	−0.014 (2)
C21	0.047 (2)	0.081 (3)	0.066 (2)	−0.004 (2)	0.009 (2)	−0.013 (2)
C22	0.047 (2)	0.056 (2)	0.059 (2)	0.0006 (18)	0.0067 (18)	−0.0029 (17)
C23	0.049 (2)	0.056 (2)	0.066 (2)	−0.0120 (19)	0.0161 (19)	−0.009 (2)
C24	0.095 (4)	0.062 (3)	0.092 (3)	−0.030 (3)	−0.003 (3)	0.005 (3)
C25	0.130 (5)	0.074 (3)	0.112 (4)	−0.036 (4)	0.028 (4)	0.002 (3)
C26	0.089 (4)	0.098 (4)	0.116 (4)	−0.045 (3)	0.030 (3)	−0.027 (3)
C27	0.052 (3)	0.113 (4)	0.141 (5)	−0.031 (3)	0.016 (4)	−0.054 (4)
C28	0.049 (2)	0.081 (3)	0.092 (3)	−0.012 (2)	0.004 (2)	−0.021 (3)
C29	0.046 (2)	0.058 (2)	0.086 (3)	0.007 (2)	−0.001 (2)	−0.003 (2)
C30	0.070 (3)	0.064 (3)	0.099 (3)	0.008 (3)	−0.015 (3)	0.008 (3)
C31	0.068 (4)	0.096 (4)	0.127 (5)	0.030 (3)	−0.036 (4)	−0.020 (4)
C32	0.045 (2)	0.087 (4)	0.123 (4)	0.010 (3)	−0.011 (3)	−0.034 (4)
C33	0.040 (2)	0.067 (3)	0.082 (3)	−0.003 (2)	0.003 (2)	−0.022 (2)
C34	0.111 (5)	0.067 (3)	0.085 (4)	−0.016 (3)	−0.021 (3)	0.007 (3)
C35	0.154 (7)	0.075 (4)	0.114 (5)	−0.020 (4)	−0.063 (5)	0.025 (3)
C36	0.137 (6)	0.082 (4)	0.131 (6)	−0.004 (4)	−0.045 (5)	0.036 (4)
C37	0.103 (4)	0.058 (3)	0.113 (4)	0.004 (3)	−0.013 (4)	0.015 (3)
C38	0.069 (3)	0.059 (3)	0.079 (3)	−0.002 (2)	−0.002 (2)	0.006 (2)

### Geometric parameters (Å, °)

**Table:**

Co1—O1	1.885 (3)	C15—C17	1.525 (5)
Co1—O2	1.891 (3)	C15—C16	1.530 (5)
Co1—N2	1.900 (3)	C15—H15	0.9800
Co1—N1	1.903 (3)	C16—C23	1.528 (5)
Co1—N4	1.967 (4)	C16—H16	0.9800
Co1—N3	1.977 (3)	C17—C18	1.374 (6)
Cl1—O5	1.381 (5)	C17—C22	1.383 (5)
Cl1—O4	1.414 (5)	C18—C19	1.368 (6)
Cl1—O6	1.421 (5)	C18—H18	0.9300
Cl1—O3	1.482 (5)	C19—C20	1.347 (7)
O1—C2	1.309 (5)	C19—H19	0.9300
O2—C13	1.322 (5)	C20—C21	1.385 (6)
O7—C39	1.53 (2)	C20—H20	0.9300
O7—H7	0.8200	C21—C22	1.407 (5)
O8—H8C	0.8501	C21—H21	0.9300
O8—H8D	0.8501	C22—H22	0.9300
N1—C7	1.291 (5)	C23—C28	1.364 (6)
N1—C15	1.492 (5)	C23—C24	1.403 (7)
N2—C14	1.284 (4)	C24—C25	1.405 (7)
N2—C16	1.483 (5)	C24—H24	0.9300
N3—C29	1.334 (6)	C25—C26	1.351 (9)
N3—C33	1.337 (5)	C25—H25	0.9300
N4—C34	1.310 (6)	C26—C27	1.362 (10)
N4—C38	1.369 (6)	C26—H26	0.9300
C1—C6	1.411 (6)	C27—C28	1.401 (7)
C1—C2	1.420 (6)	C27—H27	0.9300
C1—C7	1.441 (5)	C28—H28	0.9300
C2—C3	1.436 (6)	C29—C30	1.381 (6)
C3—C4	1.367 (6)	C29—H29	0.9300
C3—H3	0.9300	C30—C31	1.367 (8)
C4—C5	1.383 (7)	C30—H30	0.9300
C4—H4	0.9300	C31—C32	1.381 (8)
C5—C6	1.373 (6)	C31—H31	0.9300
C5—H5	0.9300	C32—C33	1.368 (7)
C6—H6	0.9300	C32—H32	0.9300
C7—H7A	0.9300	C33—H33	0.9300
C8—C13	1.412 (5)	C34—C35	1.382 (8)
C8—C9	1.416 (6)	C34—H34	0.9300
C8—C14	1.431 (5)	C35—C36	1.366 (10)
C9—C10	1.357 (6)	C35—H35	0.9300
C9—H9	0.9300	C36—C37	1.396 (10)
C10—C11	1.378 (7)	C36—H36	0.9300
C10—H10	0.9300	C37—C38	1.382 (7)
C11—C12	1.354 (7)	C37—H37	0.9300
C11—H11	0.9300	C38—H38	0.9300
C12—C13	1.416 (6)	C39—H39A	0.9600
C12—H12	0.9300	C39—H39B	0.9600
C14—H14	0.9300	C39—H39C	0.9600
			
O1—Co1—O2	87.05 (11)	N1—C15—H15	107.5
O1—Co1—N2	178.89 (13)	C17—C15—H15	107.5
O2—Co1—N2	93.08 (12)	C16—C15—H15	107.5
O1—Co1—N1	95.62 (12)	N2—C16—C23	115.1 (3)
O2—Co1—N1	177.32 (12)	N2—C16—C15	106.6 (3)
N2—Co1—N1	84.24 (13)	C23—C16—C15	112.3 (3)
O1—Co1—N4	86.43 (14)	N2—C16—H16	107.5
O2—Co1—N4	88.66 (15)	C23—C16—H16	107.5
N2—Co1—N4	92.48 (14)	C15—C16—H16	107.5
N1—Co1—N4	91.49 (14)	C18—C17—C22	118.1 (4)
O1—Co1—N3	87.91 (13)	C18—C17—C15	123.1 (4)
O2—Co1—N3	88.89 (14)	C22—C17—C15	118.7 (3)
N2—Co1—N3	93.19 (12)	C19—C18—C17	121.2 (5)
N1—Co1—N3	91.22 (13)	C19—C18—H18	119.4
N4—Co1—N3	173.94 (14)	C17—C18—H18	119.4
O5—Cl1—O4	110.2 (4)	C20—C19—C18	121.2 (5)
O5—Cl1—O6	109.3 (3)	C20—C19—H19	119.4
O4—Cl1—O6	109.2 (3)	C18—C19—H19	119.4
O5—Cl1—O3	110.5 (4)	C19—C20—C21	119.9 (4)
O4—Cl1—O3	109.2 (3)	C19—C20—H20	120.0
O6—Cl1—O3	108.4 (3)	C21—C20—H20	120.0
C2—O1—Co1	124.0 (3)	C20—C21—C22	118.8 (4)
C13—O2—Co1	121.5 (2)	C20—C21—H21	120.6
C39—O7—H7	109.5	C22—C21—H21	120.6
H8C—O8—H8D	108.3	C17—C22—C21	120.6 (4)
C7—N1—C15	119.7 (3)	C17—C22—H22	119.7
C7—N1—Co1	123.9 (3)	C21—C22—H22	119.7
C15—N1—Co1	115.3 (2)	C28—C23—C24	119.1 (4)
C14—N2—C16	123.1 (3)	C28—C23—C16	120.1 (4)
C14—N2—Co1	124.3 (2)	C24—C23—C16	120.7 (4)
C16—N2—Co1	112.6 (2)	C23—C24—C25	118.2 (5)
C29—N3—C33	117.6 (4)	C23—C24—H24	120.9
C29—N3—Co1	124.2 (3)	C25—C24—H24	120.9
C33—N3—Co1	118.2 (3)	C26—C25—C24	122.4 (6)
C34—N4—C38	116.9 (4)	C26—C25—H25	118.8
C34—N4—Co1	123.4 (3)	C24—C25—H25	118.8
C38—N4—Co1	119.6 (3)	C25—C26—C27	118.8 (6)
C6—C1—C2	119.6 (4)	C25—C26—H26	120.6
C6—C1—C7	117.5 (4)	C27—C26—H26	120.6
C2—C1—C7	122.7 (3)	C26—C27—C28	120.9 (6)
O1—C2—C1	124.9 (3)	C26—C27—H27	119.5
O1—C2—C3	117.4 (4)	C28—C27—H27	119.5
C1—C2—C3	117.6 (4)	C23—C28—C27	120.5 (5)
C4—C3—C2	120.6 (4)	C23—C28—H28	119.7
C4—C3—H3	119.7	C27—C28—H28	119.7
C2—C3—H3	119.7	N3—C29—C30	124.1 (4)
C3—C4—C5	121.1 (4)	N3—C29—H29	117.9
C3—C4—H4	119.5	C30—C29—H29	117.9
C5—C4—H4	119.5	C31—C30—C29	117.7 (5)
C6—C5—C4	120.4 (4)	C31—C30—H30	121.1
C6—C5—H5	119.8	C29—C30—H30	121.1
C4—C5—H5	119.8	C30—C31—C32	118.6 (5)
C5—C6—C1	120.7 (4)	C30—C31—H31	120.7
C5—C6—H6	119.7	C32—C31—H31	120.7
C1—C6—H6	119.7	C33—C32—C31	120.5 (5)
N1—C7—C1	125.0 (4)	C33—C32—H32	119.8
N1—C7—H7A	117.5	C31—C32—H32	119.8
C1—C7—H7A	117.5	N3—C33—C32	121.5 (5)
C13—C8—C9	119.0 (3)	N3—C33—H33	119.2
C13—C8—C14	122.4 (3)	C32—C33—H33	119.2
C9—C8—C14	118.5 (3)	N4—C34—C35	124.3 (6)
C10—C9—C8	121.7 (4)	N4—C34—H34	117.9
C10—C9—H9	119.2	C35—C34—H34	117.9
C8—C9—H9	119.2	C36—C35—C34	118.6 (6)
C9—C10—C11	118.7 (4)	C36—C35—H35	120.7
C9—C10—H10	120.6	C34—C35—H35	120.7
C11—C10—H10	120.6	C35—C36—C37	119.6 (6)
C12—C11—C10	122.1 (4)	C35—C36—H36	120.2
C12—C11—H11	118.9	C37—C36—H36	120.2
C10—C11—H11	118.9	C38—C37—C36	117.4 (6)
C11—C12—C13	121.0 (4)	C38—C37—H37	121.3
C11—C12—H12	119.5	C36—C37—H37	121.3
C13—C12—H12	119.5	N4—C38—C37	123.2 (5)
O2—C13—C8	123.2 (3)	N4—C38—H38	118.4
O2—C13—C12	119.4 (4)	C37—C38—H38	118.4
C8—C13—C12	117.4 (4)	O7—C39—H39A	109.5
N2—C14—C8	124.4 (3)	O7—C39—H39B	109.5
N2—C14—H14	117.8	H39A—C39—H39B	109.5
C8—C14—H14	117.8	O7—C39—H39C	109.5
N1—C15—C17	114.8 (3)	H39A—C39—H39C	109.5
N1—C15—C16	108.0 (3)	H39B—C39—H39C	109.5
C17—C15—C16	111.2 (3)		

### Hydrogen-bond geometry (Å, °)

**Table:**

*D*—H···*A*	*D*—H	H···*A*	*D*···*A*	*D*—H···*A*
O8—H8D···O6^i^	0.85	1.98	2.831 (14)	178
O8—H8C···O7	0.85	1.96	2.807 (19)	177
O7—H7···O2	0.82	2.08	2.897 (11)	171

Symmetry codes: (i) *x*+1, *y*, *z*.
